# Effect of Bariatric Surgery on Albuminuria in Non-Diabetic Non-Hypertensive Patients with Severe Obesity: a Short-Term Outcome

**DOI:** 10.1007/s11695-022-06091-z

**Published:** 2022-05-07

**Authors:** Ehab Fathy, Hesham Ahmed Abou Aisha, Amir K. Abosayed, Ahmed Mohammed Salah Eldeen Othman ElAnsary, Ahmad Abd Al Aziz

**Affiliations:** General Surgery Department, Kasralainy School of Medicine, Cairo University, Cairo, 12613 Egypt

**Keywords:** Obesity, Bariatric surgery, Albuminuria, No diabetes mellitus, No hypertension

## Abstract

**Background:**

Obesity is a risk factor for chronic kidney disease and albuminuria. Despite the well-documented obesity association with diabetes mellitus and hypertension, its predisposition to albuminuria is not related to these comorbidities, and, in some times, its occurrence is independent of DM or hypertension.

**Purpose of the study:**

The present study aimed to evaluate bariatric surgery effect on albuminuria in patients with severe obesity with no DM or hypertension.

**Materials and methods:**

The study consisted of 137 patients with extreme obesity and albuminuria scheduled for bariatric surgery and did not have diabetes or hypertension. They underwent an assessment for 24-h urinary albumin at baseline (T0) and 6 months postoperatively (T2).

**Results:**

Albuminuria remission occurred in 83% of patients; there was a statistically highly significant difference between the baseline and the 6-month postoperative in the 24-h urinary albumin assessment. Weight loss and BMI at T2 were independent predictors of albuminuria remission.

**Conclusion:**

The current work emphasizes the importance and promising role of bariatric surgery as an effective weight reduction management method in improving albuminuria, an early sign of chronic kidney disease, and a potential risk factor for cardiovascular disease.

## Introduction

Obesity is currently considered a pandemic. It is a common problem affecting humans in developed and under-developed countries. Worldwide, obesity-associated diseases and disorders are the leading causes of morbidity and mortality [1]. Given the limited efficacy of lifestyle modification and medications in weight reduction, bariatric surgery choice has been adopted for patients with severe obesity [2].

There is a rising interest regarding bariatric surgery in the renal community considering the promising long-term effect of bariatric surgery on hypertension, diabetes mellitus (DM) and chronic kidney disease (CKD) [3].

The obesity wide prevalence has been side by side paralleled by a CKD elevation. Obesity is now documented to be an independent risk factor for CKD. Recently, it has been estimated that obesity could be implicated in about 24–33% of kidney diseases [4].

Albuminuria has been documented to be a predictor of CKD [5], and it has been reported that albuminuria shows increased prevalence in obese individuals [6]. This has been partially explained by the obesity-induced elevated intra-abdominal pressure that leads to renal venous stasis [7]. Weight reduction via lifestyle modification or bariatric surgery has been associated with a salutary effect on albuminuria [8, 9].

Despite bariatric surgery promising outcome in ameliorating obesity and the associated comorbidities [10], there is still a lack of reports addressing its potentially beneficial effect on obesity-related albuminuria in non-diabetic, non-hypertensive patients.

Taking this in mind, the current work aimed to assess the bariatric surgery effect on albuminuria occurring in patients with severe obesity who are non-diabetic and non-hypertensive.

## Patients and Methods

This is a prospective study that included patients with severe obesity who were scheduled for bariatric surgery in Cairo University hospitals from October 2019 to February 2021. The study was conducted after the approval of the research ethics committee and in accordance with the declaration of Helsinki.

Patients indicated for bariatric surgery in our institution were those having BMI ≥ 40 kg/m^2^, aged between 16 and 65 years, and generally fit for anesthesia and surgery.

Patients who had no history of DM or hypertension underwent blood pressure measurement, assessment for 24-h urinary albumin, kidney function tests, urine analysis and HbA1c, and examination by abdominal ultrasound focused on both kidneys. Diabetes mellitus was diagnosed based on the ADA criteria (HbA1C values equal to or more than 6.5%).

Patients with asymptomatic micro or macroalbuminuria and committed to the long-term follow-up were recruited to the study.

Patients with abnormal HbA1c, urinary tract infection, and laboratory or ultrasound evidence of chronic renal impairment or accidentally discovered hypertension (blood pressure ≥ 140/90 in two different visits, 1 week apart) were excluded from the study. Also, patients on angiotensin converting enzyme inhibitors or angiotensin II receptor blockers, those with history or suggesting criteria for any systemic autoimmune diseases, and those with psychic disorders were excluded from the study.

Written informed consent was obtained from each patient after a thorough explanation of the research steps.

The operation type was selected for each patient according to the department protocol and standard indications.

Preoperative and postoperative evaluation and management were carried out in accordance with the standard strategy. It included a thorough history obtaining, relevant laboratory tests, endocrinal workup, psychological assessment, and counseling by a nutrition specialist to offer a suitable low caloric diet matching each patient’s overall condition and BMI for 1–3 weeks before surgery. All comorbidities that incur perioperative risk were controlled preoperatively as far as possible.

Bariatric surgery was conducted under general anesthesia. After pneumoperitoneum creation, the selected surgery was performed. Those were laparoscopic sleeve gastrectomy (LSG), one anastomosis gastric bypass (OAGB), or Roux-en-Y gastric bypass (RYGB).

After surgery, patients were encouraged for early mobility a few hours postoperatively. Patients were allowed for oral fluid intake on the same day of operation, and then diet was changed gradually from fluid to solid within 2 to 3 weeks. Patients were administered intravenous proton pump inhibitors (PPIs) on the day of surgery and then continued on the oral form 6 to 8 weeks after commencing oral feeding. Postoperative prophylactic anticoagulant therapy was prescribed for a period of 2 weeks. Patients were advised to proceed gradually to the regular food after 1 month of surgery except high sugar and fatty foods.

### Follow-up Assessment of the Patients

Patients who were eligible for the study underwent a follow-up by thorough history taking and clinical examination preoperatively (T0) and post operatively at 2 weeks (T1) and 6 months (T2).

At 6 months postoperatively (T2), patients were assessed for 24-h urinary albumin and urine analysis, and then patients were categorized into two groups; group A included patient with complete albuminuria remission (24-h urinary albumin ≤ 30 mg); group B included those still showing albuminuria. Patients who developed urinary tract infections postoperatively were further excluded from the study.

### Study Outcomes

The primary outcome was the difference between T0 and T2 in 24-h urinary albumin levels and the percentage of potential cases with albuminuria remission. The secondary outcome was the albuminuria remission potential predictors.

### Statistical Methods

The obtained data were recorded and analyzed using the statistical package SPSS, version 22 (IBM Corp., Armonk, NY, USA). Numerical data were presented as mean, standard deviation, minimum, and maximum, and categorical data were expressed as frequencies and percentages. Independent *t*-test was used to compare between numerical data in the two groups. Paired *t*-test was used to compare between numerical data at two-time settings. Chi-square (*X*^2^) test was used to compare between categorical data. The level of significance was considered at *p*-values less than 0.05.

## Results

The total number of patients screened during the study period was 1045. According to the inclusion and exclusion criteria, only 113 patients were eligible for the study, denoting an albuminuria prevalence of 10.8%. Eight patients dropped out and did not complete the T2 examination, and another five patients were excluded based on T2 urine analysis due to urinary tract infection. Finally, one hundred patients were the study population.

### Baseline Data of the Study Patients

The included patients’ age ranged from 21 to 61 years, with a mean of 36.93 years, and the BMI ranged from 40 to 87, with a mean of 54.17 kg/m^2^. The female gender constituted 67% of cases. The predominant indicated surgical procedure was LSG (84%).

Twenty-four–hour urinary proteins ranged from 34 to 467 mg, with a mean of 271.61 mg. According to these levels, micro-albuminuria (albumin levels of 30–300 mg/24 h) was diagnosed in 48 patients (48%), while macro-albuminuria (albumin levels of > 300 mg/24 h) was diagnosed in 52 patients (52%).

### Six-Month Postoperative (T2) Data of the Study Patients

Patients’ evaluation 6-month post-surgery (T2) revealed that BMI ranged from 29 to 55 kg/m^2^, with a mean of 35.27 kg/m^2^. The percentage of total weight loss (%TWL) ranged from 14.93 to 41.46%, with a mean of 27.61%.

### Comparison Between Baseline and 6-Month Postoperative Data of the Patients

Statistically highly significant differences were found between both time settings regarding weight, BMI, and 24-h urinary albumin (*p* < 0.001).

### Comparison Between Albuminuria in Remitted and Non-Remitted Groups

According to the 24-h urinary albumin, eighty-three patients (83%) showed albuminuria remission and constituted group A, while 17 patients (17%) were still having high 24-h urinary albumin and constituted group B. Those were 15% with micro-albuminuria and 2% with macro-albuminuria.

There were no statistically significant differences between both groups regarding the baseline or the postoperative criteria, apart from the weight loss amount at T2 (*p* = 0.012) (Table 1).

**Table 1:** Comparison between albuminuria remitted and non-remitted cases at 6-month postoperative (T2)

		Group A		Group B		*t*	*p*
		Mean ± SD		Mean ± SD			
Age (years)		36.5 ± 10.3		39 ± 6		− 0.97	0.34
Height (m)		1.6 ± 0.1		1.7 ± 0.7		0.63 −	530
Weight at T0 (kg)		152.3 ± 24.6		140.7 ± 19.5		1.8	0.07
Weight at T2 (kg)		109.9 ± 21.4		105 ± 16.7		0.89	0.37
BMI at T0 (kg/m^2^)		54.6 ± 10.2		51.9 ± 9.5		1.04	0.3
BMI at T2 (kg/m^2^)		34.9 ± 4.9		37 ± 5		1.59 −	0.12
24-h urinary proteins at T0 (mg)		277.4 ± 137.3		243.5 ± 155.5		0.91	0.37
24-h urinary proteins at T2 (mg)		16.2 ± 6		152 ± 102.5		− 12.1	0.00
T2 weight loss (kg)		42.4 ± 9.7		35.7 ± 10.3		2.57	0.012*
%TWL		28.1 ± 5.5		25.3 ± 6.4		1.84	0.07
		Count	%	Count	%	*X*^2^	*p*
Gender	Female	57	57	10	10	0.62	0.43
	Male	26	26	7	7		
Type of surgery	LSG	70	70	14	14	1.62	0.44
	RYGB	1	1	1	1		
	MAGB	21	21	2	2		
Albuminuria at T0	Macro-albuminuria	44	44	8	8	0.2	0.65
	Micro-albuminuria	93	93	9	9		

### Factors Predicting Albuminuria Remission

A model containing the postoperative weight measures showed that T2 BMI and T2 weight loss were the predictors for albuminuria remission, with *p* values of 0.029 and 0.010, respectively (Table 2).

**Table 2:** Binary logistic regression analysis including postoperative weight measures for predicting albuminuria remission

Model	B	S.E	*p*
T2 weight	0.02	0.02	0.2
T2 BMI	− 0.13	0.06	0.029*
T2 weight loss	0.08	0.03	0.01*
Constant	0.89	2.23	0.69

*B*, the slope of regression line; *S.E*, the standard error. *: statistically significant

## Discussion

Obesity has been reported to be a risk factor for chronic kidney disease and albuminuria [4]. Despite the well-documented obesity association with diabetes mellitus and hypertension [11], its predisposition to albuminuria is only partially related to these comorbidities, and, in some times, its occurrence is independent of DM or hypertension [12]. Some studies related micro-albuminuria to a poor outcome of cardiovascular diseases as well as being an alarming sign for the development of chronic kidney disease [13]. Scarce data about the prevalence of albuminuria in patients with obesity in the absence of DM and hypertension are available. Moreover, the effect of bariatric surgery on albuminuria in patients having it without DM or hypertension was not previously reported.

The present study aimed to evaluate the effect of bariatric surgery on albuminuria occurring in patients with severe obesity and having no DM or hypertension.

In this study, the albuminuria prevalence was 10.8%. This is close to the figures reported by earlier studies; studies from France and USA found that albuminuria occurred in obese patients without DM and hypertension, with percentages of 9.8% and 10%, respectively [14, 15]. Another study from the Netherlands reported the albuminuria prevalence in patients with severe obesity to be ranging from 13 to 21%. However, about half of these cases had hypertension [16]. The prevalence found in this study was higher than reported by another Egyptian study that reported albuminuria prevalence of 6.5% in patients having obesity without DM or hypertension. This variation is mainly caused by the difference in the study population since their study included individuals with a BMI value ranging from 25 to less than 35 kg/m^2^ [17].

The patients showed a highly significant reduction in weight and BMI after 6 months of surgery in the current study. This ensures the well-documented weight reduction effect of bariatric surgery [18, 19].

Regarding the primary outcome of the current study, the 24-h urinary albumin levels were significantly reduced 6 months postoperatively compared to the preoperative levels, with observed albuminuria remission in 83% of the patients.

The previous studies assessing the bariatric surgery effect on patients with albuminuria included patients with DM, hypertension, or even both. Most of these studies reported a reduction in urinary albumin levels (6–24 months postoperatively) compared to the baseline levels [20, 21].

According to the previous studies, the albuminuria reduction was explained by improving glucose metabolism, blood pressure, and systemic inflammation [22]. Improving systemic inflammation was specified to be the underlying cause in other studies [23, 24]. This was mirrored by decreased levels of renal cytokines [25] and increased levels of the anti-inflammatory adipokine adiponectin [12]. Given our exclusion of cases having abnormal blood pressure and blood glucose levels, systemic inflammation improving or relieving the pressure induced by the excess abdominal adipose tissue may be the cause of albuminuria reduction in our study. However, it is still uncertain whether albuminuria reduction is attributed to a reduction in weight or the improvement in systemic inflammation elicited by a reduction in weight.

Regarding the secondary outcome in this study, the study groups differed significantly only in the weight loss amount at T2. This was further affirmed by the regression analysis, which revealed that the postoperative BMI and weight loss were predictors for albuminuria remission.

Within the same context, Amor et al. [26] found that the reduction in weight appeared to be an independent contributing factor to the normalization of albumin-creatinine ratio (ACR) in patients with severe obesity undergoing bariatric surgery. They reported that the BMI changes from baseline values were an independent predicting factor for normal ACR at 12 months. However, in contrast to our findings, Park et al. [27] found that hs-CRP was the independent risk factor predicting ACR, while body weight and BMI were not.

Another aspect of the inverse association between albumin and obesity has been recently explored. Despite being historically considered a protein malnutrition marker, a recent focus on albumin as an inflammatory marker has been adopted [28]. The association between low albumin levels and a subclinical inflammatory state was argued to be explaining the link between albumin and obesity [29]. Moreover, it was found that albumin binds ghrelin, impacting its orexigenic effects and contributing to appetite regulation [30]. It seems like a vicious circle in which weight reduction improves the albuminuria state, hence, improves the serum albumin levels, which, in turn, enhances the weight reduction.

The data above emphasize the beneficial effects of bariatric surgery on the overall health status, rather than just a weight-decreasing procedure.

## Strength and Limitations

The strength in this study is being a prospective longitudinal study, evaluating patients at two-time settings, and specifying patients without DM and hypertension to ensure that the studied disorder is related to obesity itself as far as possible, and that the resultant bariatric surgery effect is mainly attributed to weight reduction, either directly or indirectly. The study is limited by the non-assessment of the inflammatory markers or the anti-inflammatory adipokine adiponectin to assess the potential systemic inflammation mediating role in the albuminuria reduction effect. Also, it is limited by the short follow-up period of 6 months. However, we think that these limitations do not preclude the importance of the study.

## Conclusion

The current work emphasizes the bariatric surgery importance and promising role as an effective weight reduction management method in improving albuminuria, an early sign of chronic kidney disease and a potential risk factor for cardiovascular disease.
